# “I feel good in this creative world”: a multi-methods study exploring older artisans’ attributions of mental health, quality of life, and well-being to their work in a UNESCO Creative City of crafts and folk art

**DOI:** 10.3389/fpubh.2025.1651932

**Published:** 2025-10-02

**Authors:** Sandra Igreja, Constança Paúl, Soraia Teles

**Affiliations:** RISE-Health, Department of Behavioral Sciences, School of Medicine and Biomedical Sciences, University of Porto (ICBAS-UP), Porto, Portugal

**Keywords:** mental health, psychological well-being, work, older adults, art, crafts, generativity

## Abstract

In the face of global demographic shifts, it is crucial to adopt sustainable solutions that support active and healthy aging. Engagement in creative activities, such as crafts, may positively affect the social determinants of health by promoting mental, physical, and social well-being. While the use of crafts as therapeutic interventions is well documented, research on crafts as a professional activity remains limited, especially among older adults and across different sectors. This multi-methods study combined quantitative data analysis with qualitative exploration of individual narratives to examine how older professional artisans in a UNESCO Creative City of Crafts and Folk Art in Portugal perceive the impact of craftwork on their health and well-being. Fifty-five artisans aged 55 and older took part in walking interviews in their ateliers and completed health and quality of life (QoL) scales (PHQ-8 and WHOQOL-BREF). Thematic content analysis was performed by two independent coders. Participants were 60% men (*n* = 33) and had a mean age of 67.5 years (SD = 8.02). Each participant was active in one of eight craft sectors represented in the study. The artisans had overall good QoL scores, with the psychological domain showing the highest average (WHOQOL-BREF M = 84.92, SD = 10.98). All but one participant (98.1%) scored below 10 on the PHQ-8, indicating the absence of depressive symptoms. Thematic analysis revealed artisans’ perceptions of both positive and negative impacts of craftwork on their well-being, with 17 areas of impact emerging from their discourses. Positive attributions predominated, with craftwork being associated with psychological well-being, personal and professional fulfilment, creative identity expression and a sense of generativity. In contrast, financial instability and labor precarity emerged as the main strains of craftwork. The study suggests that older artisans understand their professional involvement in crafts and folk art as contributing to their well-being, even in the face of economic challenges associated with this work. These results support policy recommendations, aimed at improving the economic and working conditions of artisans, as well as knowledge transmission programs as strategies to promote active and healthy aging, foster intergenerational learning, and preserve intangible cultural heritage.

## 1 Introduction

In light of the demographic changes occurring globally, it has become a priority to identify solutions that support active and healthy aging, while recognizing the role of older individuals in society (1–3).

Among the possible strategies, participation in productive, creative, and socially meaningful activities stands out as a way to promote the health and well-being of older adults (4–10).

Previous studies have observed that working longer into later life may offer health benefits (11–13), and that professional activity is an important factor in the quality of life for older individuals (13, 14). Well-being, particularly in its dimensions of meaningfulness of society and personal growth, has been associated to being employed in later life (15). However, this association may depend on the type of occupation, job characteristics, and sociocultural contexts (15). Reflecting this context-dependence, for some men and women, post-retirement employment holds added value, particularly due to its positive effects on health (16).

This study investigates well-being in the context of artistic work. Research on work and well-being has been widely conducted with professionals from various artistic fields, such as visual arts, dance, theater, and music. For example, studies have examined the characteristics of work and health among performing artists (17); others have focused on orchestral musicians (18), as well as on the symbolic constructions developed by professionals in areas such as music, theater, and dance (19). Additional studies have identified factors related to the well-being of professionals in the visual, musical, sonic, and performing arts, encompassing fields such as fine arts, graphic design, animation, film, photography, dance, and theater (20, 21). Moreover, several studies indicate that art serves as an effective therapy in reducing depressive symptoms (22–24). Despite this body of literature, few studies have addressed arts and crafts practices, especially among older individuals (8, 25, 26). Previous research has explored how artisans describe the meaning of craft as a practice associated with their well-being, indicating that this activity may promote well-being both through the artifacts produced and through the sense of fulfillment derived from the creative process (27). There are several factors that contribute to the meaning of work, including personal and contextual aspects (28). Moreover, other studies have observed that motivational processes play a crucial role in experiencing a meaningful life, influencing how this experience is perceived (29). In this context, generativity, understood as a central task of the adult life cycle, emerges as an important dimension of motivational processes, reflecting concern for contributing to future generations. Generativity integrates personal and cultural goals, the nature of which must be understood within each adult’s life, unique in their desires, concerns, beliefs, and commitments, situated within a specific social and historical context (30, 31).

The relevance of the arts to well-being is also recognized by major health organizations. The World Health Organization has historically integrated the arts into health promotion, emphasizing that physical, mental, and social well-being go beyond the mere absence of disease. The creation and appreciation of the arts are seen as contributing to overall well-being, social inclusion, and quality of life throughout the life course (32). The literature has widely documented the mental health benefits of engaging in artistic practices (7, 22, 24, 33–35).

Numerous individual health benefits have been described for both professional and amateur artists, such as enhanced emotional well-being, increased life satisfaction, and cognitive engagement (8, 26). However, significant gaps remain in the literature, particularly regarding arts and crafts practices, which have been less extensively studied compared to other art forms like music and dance (8, 25, 26).

Craft practices promote both physical and mental stimulation, encourage creativity, and support social interaction, including intergenerational connections (36). Many individuals are emotionally invested in craftwork and maintain this practice throughout all stages of life (36). Craft is often described as a well-being promoting occupation, with impacts on personal growth, the development of physical and cognitive skills, bodily and emotional control, and the strengthening of cultural and social awareness (27), as evidenced by studies conducted in the field of textile crafts. Beyond immediate and hedonic pleasure, this practice contributes to psychological well-being and a balanced life in the long term (6), based on findings from the same field.

Artistic practice has demonstrated important benefits, including emotional regulation and stimulation of brain plasticity, promoting lifelong learning (37). Lifelong learning has contributed to expanding the concept of active aging, reinforcing its original pillars and recognizing the essential role of information in this process (38–40). Additionally, engagement with creative arts, including crafts, is accessible and popular among the general population, highlighting its potential as a relevant tool for public health promotion (9).

In Portugal, studies indicate that physical and emotional conditions influence self-assessed health and life satisfaction, with income being a factor that has a direct impact on this perception, as well as acting as a mediator through self-perceived health (41). Previous studies indicate that positive feelings predict longevity and health beyond negative feelings, emphasizing the importance of emotional well-being in aging populations (42).

There is a need to build further knowledge about the effects of the arts on the health and quality of life of older adults (25), and research on spontaneous artistic activities carried out in natural contexts (i.e., not derived from therapeutic interventions) remains limited (6, 26, 43, 44). In particular, activities such as arts and crafts remain understudied, despite recognition in the literature of the need for further research in this area (8, 26, 34). Beyond the underrepresentation of arts and crafts, other gaps have been identified in the literature on older adults’ engagement in artistic activities, including the lack of an approach that values the inclusion of older adults’ own voices and their diversity, as well as the lack of a contextualized approach to these practices (26). Studies examining the effects of arts involvement in healthy populations also point to a lack of qualitative evidence to deepen the understanding of the role of artistic engagement and to reveal how participants experience these activities (5).

In this context, the present study gains relevance by focusing on a UNESCO Creative City, internationally recognized for its crafts and folk art. This approach may broaden the discussion beyond the local scope, connecting it to global debates on active and healthy aging, culture, and sustainable development, aligning with current international agenda and guidelines (2, 3, 45–47).

A previous study, conducted by this research team, described the perceptions of health, quality of life, and happiness among older professional artisans from a UNESCO Creative City of Crafts and Folk Art in Portugal (Barcelos), and examined its associations with sociodemographic and professional variables (48). Although this quantitative study revealed positive indicators of health, quality of life, and well-being, it did not explore the extent to which these results were attributable to engagement in crafts and folk art from the artisans’ perspective. The present study aims to explore how older professional artisans in this Creative City perceive the impact of their activity in crafts and folk art on their health, quality of life, and well-being.

Therefore, in the present study, the concept of attribution is central, referring to how individuals explain the causes of their behaviors or others’ behaviors, which can be seen as internal or external factors. This process helps understand how people relate their actions to outcomes, influencing their emotional responses and future motivations (49–51).

The context of this study is particularly interesting as the region (Barcelos) is deeply shaped by its artisanal identity, characterized by a rich variety of arts and crafts, with particular prominence in pottery and the crafting of clay figures. Barcelos hosts numerous ateliers dedicated to various sectors of crafts and folk art, including: Imagery, Pottery, Embroidery, Weaving, Iron and Derivatives, Wood, Basketry and Wicker, and Contemporary Crafts, designated as a UNESCO Creative City of Crafts and Folk Art (52, 53). The city was also designated as the first World Mental Health Capital by the World Federation for Mental Health, in recognition of its contributions and commitment to the mental health cause (54, 55). Hence, this setting - with its strong artisanal identity and significant public investment in preserving the cultural heritage of arts and crafts - offers a unique opportunity to explore the research questions posed in this study.

This study is part of a larger project aimed at exploring the contribution of artistic activity and the legacy of crafts and folk art to the quality of life, health status, and family cohesion of professional artisans working in Barcelos (Portugal).

## 2 Materials and methods

### 2.1 Study design

An observational, cross-sectional, multi-methods study was conducted, considering sociodemographic and professional data alongside quality of life and psychological well-being scales, together with the walking interview method to collect primary data in the ateliers of older professional artisans. This approach enables methodological triangulation, enriching the understanding of the relationships between craft activity, health, and well-being.

Triangulation, defined as the combined use of qualitative and quantitative methods to study the same phenomenon, is seen as a strategy to increasing the validity of the results and contributing to theory and knowledge development (56, 57).

In methodological triangulation, the central question is whether the theory guiding the investigation is developed inductively from the data themselves or applied deductively (56). Content analysis should be conducted inductively, allowing categories to emerge from the data without forcing preconceived frameworks to fit the quantitative analysis (56). In this study, the qualitative data, collected until saturation, was analyzed inductively. This approach allowed categories to emerge from the data without forcing preconceived frameworks to fit the quantitative analysis. Quantitative data was analyzed in a descriptive approach. The complexity of reality supports an alternative epistemological stance in which various methods, originating from different epistemological traditions, when combined, add new perspectives to the phenomenon under study (58).

### 2.2 Participants and recruitment

Data were collected from professional artisans aged 55 or older, who were actively working in ateliers located in Barcelos. This was a non-probabilistic convenience sample covering different craft sectors in the region. Artisans from any of the following craft sectors were eligible: Imagery, Pottery, Embroidery and Weaving, Iron and Derivatives, Wood, Basketry and Wicker, and Contemporary Crafts. In addition to regular activity in the sector, participants were required to have their atelier located within the territory of Barcelos (Portugal) and to reside in the community (not in institutional care). Exclusion criteria included users of day care or social centers, as they receive regular support from institutions and do not fit the profile of active, community-based professional artisans.

Potential participants were identified through public platforms, namely the official website of the Municipality of Barcelos, complemented by the websites of artisans with an online presence. This municipal website provides information on the craft routes (a comprehensive mapping of artisans, including the location of their ateliers and contact details) by craft sector (52), which enabled contact with artisans from different sectors within the municipality. The initial contact was made by telephone, during which artisans were provided with comprehensive information about the study. All artisans who met the eligibility criteria were invited to participate, and subsequent data collection steps were scheduled with eligible participants according to their availability. Data collection took place in the artisans’ ateliers during the first quarter of 2024 and continued until data saturation was reached, that is, when no new themes emerged and the information became redundant across the in-depth interviews conducted in the ateliers.

This study received a favorable opinion by the Ethics Committee of the Centro Hospitalar Universitário de Santo António, E. P. E. (CHUdSA) and the Scholl of Medicine and Biomedical Sciences, University of Porto (ICBAS-UP) [CHUdSA/ICBAS Ethics Committee] [reference 2024/CE/P02 (P418/2023/CETI)]. It was approved by the Data Protection Unit of the University of Porto (reference R-6/2025) and conducted in accordance with local legislation and institutional requirements.

All participants were provided with detailed information about the study through a “Participant Information in the Study,” which explained the research objectives, participation conditions, data confidentiality, potential benefits and risks, and contact details for further inquiries. Participation was voluntary, with no financial incentives or compensation offered. Informed consent was obtained in writing from all participants prior to data collection, ensuring they fully understood the nature and objectives of the study.

### 2.3 Instruments

A survey specifically designed to collect sociodemographic information, data on professional activity, and aspects related to health, quality of life, and well-being was administered to the study participants. Sociodemographic information included the age, gender, marital status, years of education, primary source of income, monthly income, and length of residence in the locality. Details on their professional activity included professional training, professional status, artistic sector of activity, age of entry into the activity, main raw materials used, predominant themes represented, weekly hours dedicated to the activity, and the days of the week they engage with it.

To assess participants’ perceptions of quality of life and health status, the European-Portuguese version of the WHOQOL-BREF scale was administered (59, 60). This instrument comprises 26 questions, two of which address overall perceptions of quality of life and health, while the remaining 24 are organized into four domains: physical, psychological, social relationships, and environment. Responses are recorded on a five-point scale, and higher scores for each domain indicate a better quality of life.

The Portuguese version of the WHOQOL-BREF shows good psychometric properties, with high internal consistency for the 26 items (*α* = 0.92) and Physical (*α* = 0.87), Psychological (*α* = 0.84), Environment (*α* = 0.78), and Social Relationships (*α* = 0.64) domains (60).

In addition, the Patient Health Questionnaire PHQ-8 scale (61)was used to assess the presence of depressive symptoms (61). Comprising eight items, each is rated from zero to three depending on the frequency of the symptoms, which ranges from “not at all” to “nearly every day,” with the total score ranging from zero to twenty-four (61, 62). Current depression can be defined using a cutoff score ≥ 10 (61). The PHQ-8 has shown good sensitivity and specificity for identifying depressive disorders (63). This instrument has also been used in the Portuguese National Health Survey (64).

To assess the degree of happiness with life, a question from the European Survey on Aging Protocol (ESAP) was used: “Compared to other people and considering the balance between the good and bad events in your life, to what extent do you feel well and happy at this moment?” (65). The response is given on a scale from one to four, with higher scores indicating greater happiness.

For the interviews conducted in this study, the “Go-along Walking Interview” method was adopted as it is highly useful for obtaining contextualized information (66). This method is one of the walking interview formats described in the literature (66–72). Walking interviews are increasingly used in the field of health and well-being to understand the relationship between individuals and places (66, 70, 73).

A semi-structured format was adopted, with open-ended questions developed in European Portuguese, based on an interview guide previously created from the literature [e.g., (74–77)]. The guide included primary and secondary questions, asked flexibly according to the flow and spontaneous discourse of the participants, allowing the exploration of relevant emerging themes during the interviews.

The interview guide focused on: (a) the participants’ path in craft and folk art; (b) the feelings of belonging to the territory; (c) what most characterizes their artistic work; (d) whether the activity comes from previous generations and if there are more people in the family involved in the professional sector; (e) whether the start of their craft activity was spontaneous or influenced by someone else; (f) to what extent they had the opportunity to help prepare younger generations, family members or others, for the sector’s activity; (g) what it means to have had or not had this opportunity; (h) to what extent the artistic activity has influenced family relationships; (i) what causes younger generations to approach or distance themselves from the activity in the sector; (j) to what extent they feel good about life and consider themselves to have quality of life; (k) how satisfied they are with their health; (l) how the artistic activity affects (positively and/or negatively) their quality of life and well-being; (m) whether the activity contributes to personal satisfaction/self-esteem; (n) what motivates them to continue their work in the sector; (o) what they value most in the work they do; (p) whether they plan to stop working or making pieces; (q) whether they consider what they do to be relevant; (r) whether they have noticed changes over the years regarding the creative meaning of their activity; (s) whether there have been changes over the past few years in how they perform their work or how they view crafts and folk art; (t) whether they actively participate in local, national, or international dynamics, and if they participate in events related to craft and folk art; (u) whether they engage in other social participation activities; (v) what the artistic activity they perform means to them.

Field notes were documented throughout the interviews, which were audio recorded (1–2 h per interview), transcribed verbatim, and supplemented with observations/field notes.

A transcription approach that values contextual and non-verbal information was adopted to deepen the attributions to the narrated events. A repeated listening of the full recording (at least twice) was conducted to familiarize with the content and discursive particularities of the participants, noting indications considered useful for the writing and analysis of field notes, which were part of the analytical process. For accuracy of the transcribed information, the text was reviewed. All personal information identifying the participants was removed, preserving the relevant content while safeguarding the anonymity of individuals.

### 2.4 Data analysis

#### 2.4.1 Statistical analysis

For the analysis of numerical data, SPSS software, version 29, was used. Descriptive statistics were calculated including absolute and relative frequencies, measures of central tendency (e.g., mean, median) and measures of dispersion (e.g., standard deviation, variance), selected according to the nature and distribution of the data.

The original scores for each domain of the WHOQOL-BREF were transformed into a scale from 0 to 100 to facilitate interpretability, in accordance with the scoring guidelines (59, 60).

#### 2.4.2 Content analysis

A thematic content analysis of the textual data was conducted following a horizontal scheme (78). NVivo software was used (Release 1, 2020).

Data saturation has been recognized as a general guideline for determining sample size in qualitative research. To monitor data saturation, preliminary observation was conducted simultaneously with data collection. Data saturation was reached when no new themes emerged, and redundancy was perceived in participants’ responses. An additional interview, planned in advance during quantitative data collection and conducted shortly after the saturation point, was also considered to verify the maintenance of saturation. Following this interview, no further recruitment efforts were made. At this stage, the percentage of participants was mapped in comparison to the demographic reality of the territory.

Data were grouped into categories through the breakdown of the text into meaning units. The categories/subcategories and the final coding tree were defined based on a bottom-up, data-driven inductive approach. The main categories, also referred to as ‘parent’ nodes, were established as broad attributions of the participants’ perceptions. These main categories were further subdivided into ‘child’ nodes, or subcategories, detailing the specific aspects of the identified attributions.

To enhance the validity of the study, two researchers (researcher A and B) independently coded all text sources and suggested titles and definitions for categories and sub-categories.

Researcher B was not involved in the participants’ recruitment, data collection, or transcription of the interviews. The inter-rater agreement was qualitatively appreciated, and a consensus method was employed. Both researchers shared and revised their own and the other’s analysis to decide whether to change their conclusions on the categories where text excerpts were initially coded. Several iterations between researcher A and B were carried out to refine the classification tree as well as the definitions linked to each category and sub-category. Based on the agreement, a final codification tree was reached.

Several procedures were conducted during the thematic content analysis, including cross-reference tables, word clouds, and hierarchy charts. The results regarding the attributions to crafts and folk art activities among older professional artisans are presented as follows: (a) absolute frequencies of references coded by node/(sub)category; (b) absolute frequencies of artisans coded within a node/(sub)category; and (c) percentages of the source content coded within each node/(sub)category. Word frequency is presented in a word cloud. Text excerpts are included to illustrate categories.

The qualitative analysis process, conducted using a thematic content analysis approach, is described in detail in Table 1, which sequentially presents all the steps carried out from data collection to the interpretation and synthesis of the results.

**Table 1:** Steps of the qualitative analysis based on thematic content analysis.

Steps	Description of the steps of qualitative data collection and analysis
1	Data collection in participants’ ateliers through context-based interviews (Go-along Walking Interview).
2	Organization of the collected data.
3	Pseudonymization of cases.
4	Repeated listening of the full recordings to familiarize with the content and participants’ discursive particularities, with notes and field observations recorded.
5	Verbatim transcription of audio-recorded interviews (445 pages; 213,897 words), excluding field notes.
6	Ensuring confidentiality through pseudonyms and removal of all personally identifiable information while preserving relevant content and preventing participant recognition.
7	Review of transcriptions to ensure accuracy of information.
8	Reading and rereading transcriptions for in-depth familiarization with the data.
9	Inputting data into NVivo software.
10	Initial coding of sources.
11	Independent coding of all sources by two coders, with suggestions for titles and definitions of categories and subcategories.
12	Cross-review between coders of their own analyses and each other’s analyses, deciding on changes to categories in which text excerpts were initially coded.
13	Interactions between researchers A and B to refine the coding tree and definitions of each category and subcategory.
14	Based on consensus among independent coders, consolidation of the final coding tree, containing 1,579 coded text units.
15	Triangulation of qualitative data with quantitative data.
16	Development of cross-reference tables.
17	Production of hierarchy charts.
18	Thematic interpretation and synthesis of results.

The consolidated criteria for reporting qualitative research were followed (79) (see Supplementary material 1).

## 3 Results

**Figure 1:**
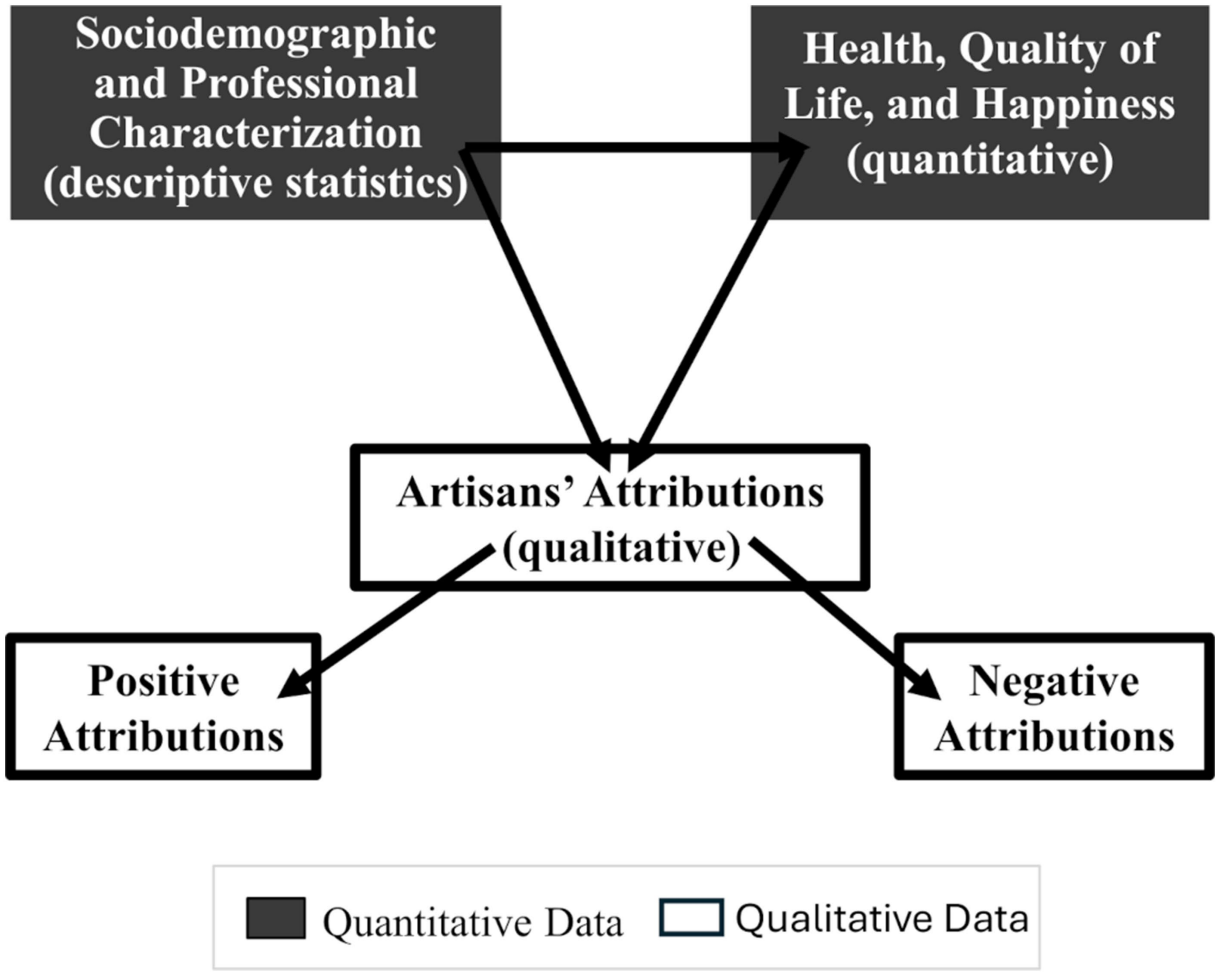
Visual representation of the interrelationship between quantitative and qualitative data from the artisans.

In this section, we present the results organized into three sections: 3.1 Characterization of the study participants (quantitative data), 3.2 Health, quality of life, and happiness of the artisans (quantitative data), and 3.3 Artisans’ attributions of health, quality of life, and well-being to their craftwork (qualitative data). Figure 1 schematically illustrates the interrelationship among these result sections.

### 3.1 Characterization of the study participants (quantitative data)

#### 3.1.1 Artisans’ sociodemographic and professional characteristics (descriptive statistics)

The study included 55 artisans, representing 27.36% of the artisan population in the study territory (Barcelos). Table 2 presents the sociodemographic and professional characteristics of the artisans.

**Table 2:** Sociodemographic and professional characteristics of participants.

Variables	N	Descriptive statistics
Sociodemographic characteristics		
Gender, n (%)	55	
Male		33 (60)
Age (years), M (SD)	55	67.49 (8.02)
Years of education, Md	55	5
Marital status, n (%)	55	
Married		47 (85.5)
Widow(er)		5 (9.1)
Divorced		2 (3.6)
Single		1 (1.8)
Professional characteristics		
Professional training, n (%)	55	
Yes		13 (23.6)
Main professional activity, crafts, n (%)	55	
Yes		49 (89.1)
Retired, n (%)	55	
Yes		33 (60)
Main source of income, n (%)	55	
Crafts		54 (98.2)
Pension/Retirement		32 (58.2)
Other income		6 (10.9)
Monthly income, based on the National Minimum Wage (NMW), n (%)	55	
Monthly income ≤ NMW		35 (63.6)
Monthly income > NMW		20 (36.4)
Years of residence in the parish, M (SD)	55	56 (19.50)
Age of entry into the craft sector, n (%)	55	
Up to 10 years old		24 (43.6)
11–20 years old		18 (32.7)
21–40 years old		4 (7.3)
41–60 years old		8 (14.6)
Over 60 years old		1 (1.8)
Craft sector, n (%)	55	
Imagery		29 (52.7)
Pottery		7 (12.7)
Wood		5 (9.1)
Iron and derivatives		4 (7.3)
Embroidery		3 (5.5)
Contemporary crafts		3 (5.5)
Weaving		2 (3.6)
Basketry and wicker		2 (3.6)
Atelier location at the residence, n (%)	55	
Yes		48 (87.3)
Weekly hours dedicated to craft activity, Md	55	50
Weekly hours dedicated to non-professional activities, Md	55	8

Most participants were male (60%, *n* = 33), married (85.5%, *n* = 47) and ages ranged from 55 to 88 years (M = 67.49, SD = 8.02).

The average number of years in formal education among the artisans was 6.42 (SD = 3.32; range 0–19 years). Professional training in various areas was reported by 23.6% (*n* = 13) of participants.

Most participants were retired (60%, *n* = 33), and 89.1% (*n* = 49) considered craftwork to be their primary occupation. For nearly all artisans (98.2%, *n* = 54), the main source of income was from their craftwork. Earnings equal to or below the national minimum wage were reported by 63.6% (*n* = 35) of participants.

Almost all participants (96.4%, *n* = 53) were homeowners, with 87.3% (*n* = 48) having their ateliers in their homes, and the median length of residence in the parish was 62 years.

Participants began their engagement in craft and folk art activities between the ages of 4 and 63 (M = 18.65 years, SD = 16.10).

The artisans’ work spans eight sectors with the Imagery one being the most represented (52.7%, *n* = 29). The median weekly time dedicated to craftwork was 50 h (range 18–96).

### 3.2 Artisans’ health, quality of life and happiness (quantitative data)

Table 3 presents the results from the mental health (PHQ-8), quality of life (WHOQOL-BREF), and happiness (ESAP) scales. Scores bellow the cutoff point for depression (< 10 on PHQ-8) were obtained by almost all participants (98.1%, *n* = 54), with only one scoring above the threshold.

**Table 3:** Health, quality of life, and happiness among older professional artisans (*n* = 55).

Variables	N	Descriptive statistics
PHQ-8 Patient Health Questionnaire n (%)	55	
Without depression (< 10)		54 (98.1)
With depression (≥ 10)		1 (1.8)
Satisfaction with health*, n (%)	55	
Dissatisfied		3 (5.5)
Neither satisfied nor dissatisfied		19 (34.5)
Satisfied and very satisfied		33 (60)
Psychological QoL^#^, M (SD)	55	84.92 (10.98)
Social Relationships QoL^#^, M (SD)		80.76 (12.61)
Environment QoL^#^, M (SD)		78.81 (9.48)
Physical QoL^#^, M (SD)		78.64 (11.91)
Overall QoL^†^, M (SD)		68.64 (13.58)
Perceived happiness^¥^, n (%)	55	
Very happy		31 (56.4)
Happy		23 (41.8)
Somewhat unhappy		1 (1.8)

*Assessed with WHOQOL-BREF, item 2. ^#^Assessed with WHOQOL-BREF; transformed scores for each domain are presented. ^†^Assessed with WHOQOL, sum of items 1 and 2, transformed score. ^¥^Assessed with one item from the European Survey on Aging Protocol (ESAP).

Regarding general health perception (Question 2 WHOQOL-BREF) most participants (60%, *n* = 33) reported being satisfied or very satisfied with their health. A similar pattern was observed for general quality of life perception (Question 1 WHOQOL-BREF), with most artisans (61.8%, *n* = 34) rating it as “good.”

Among QoL domains, the psychological one had the highest average transformed score (M = 84.92; SD = 10.98), followed by the social relationships domain (M = 80.76; SD = 12.61), the environment domain (M = 78.81; SD = 9.48), and the physical health domain (M = 78.64; SD = 11.91). The mean overall perception of quality of life (Questions 1 and 2) was 68.64 (SD = 13.58).

Most artisans (56.4%, *n* = 31) reported feeling “very happy” with their life.

### 3.3 Artisans’ attributions of health, quality of life, and well-being to craftwork (qualitative data)

The walking interviews conducted in the artisans’ ateliers resulted in 445 transcribed pages/213897 words, excluding field notes. Overall, 1,579 text units were coded.

**Figure 2:**
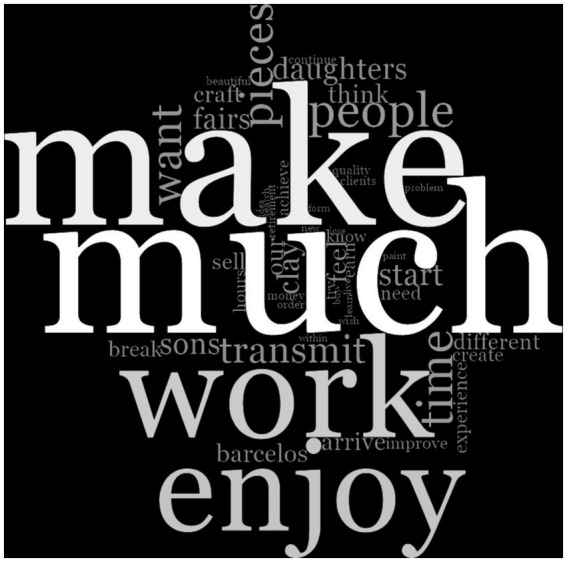
Word cloud representing the 50 most frequent words from text units describing the attributions of crafts and folk art activities.

A frequency analysis was conducted to visualize the 50 most frequent words in the participants’ discourses regarding their attributions of health, quality of life, and well-being to craft and folk art activity (Figure 2). The analysis was performed by assuming stemmed words (e.g., work, working) to avoid overlap. Functional words (e.g., “in,” “for,” “therefore”) were added to the stop words list in NVivo to be filtered out of the word count.

The word cloud shows that among the most frequent terms, several may reflect recurring themes in how participants describe their experiences with craftwork. Some words appear to relate to the practical aspects of engaging in the activity (e.g., “make,” “much”), while others may be associated with emotional or motivational language used by participants (e.g., “work,” “enjoy,” “want,” “feel,” “need”). Other emerging words are directly related to the practice of craft and folk art, as well as to territory and the dimension of time (“pieces,” “clay,” “craft,” “paint,” “Barcelos,” “time,” “hours”). Words also appear that are associated with the transmission of knowledge and family (“transmit,” “sons,” “daughters”), interaction with others and the economic dimension of the activity (“people,” “fairs,” “sell,” “clients,” “money,” “order”), as well as creativity, experience, learning, and growth (“create,” “different,” “experience,” “achieve,” “arrive” [In the sense of reaching a goal or achieving growth], “improve”).

The final coding tree resulted in a structure composed of two “parent” nodes or main categories - positive attributions (N1) and negative attributions (N2) - and seventeen “child” nodes or subcategories which represent the different types of attributions (eleven associated with positive attributions and six with negative attributions) (Table 4).

**Table 4:** Coding tree and category definitions.

Category	Description
N1_Positive Attributions	The text units in this category refer to positive attributions of health, quality of life, and/or well-being to craftwork.
N1.1_Positive implications for psychological well-being and mental health	Text units in this category refer to the attribution of increased psychological well-being and mental health benefits to craftwork.
N1.2_Occupational benefit	Text units in this category refer to the attribution of the benefit of staying active and occupied to craftwork.
N1.3_Professional fulfillment	Text units in this category refer to the attribution of professional fulfillment to craftwork.
N1.4_Realization of creative potential and creative identity	Text units in this category refer to the attribution of the realization of creative potential and creative identity to craftwork.
N1.5_Evolution and mastery	Text units in this category refer to the attribution of a sense of evolution and mastery to craftwork.
N1.6_Sense of generativity and pleasure in teaching	Text units in this category refer to the attribution of a greater sense of generativity and pleasure in teaching to craftwork, expressed through the transmission of knowledge and skills, particularly to the younger generations, but also to the community.
N1.7_Economic benefit	Text units in this category refer to the attribution of an improved economic situation to craftwork.
N1.8_Autonomy and work flexibility	Text units in this category refer to the attribution of autonomy and work flexibility to craftwork, including the freedom to decide what to do and when to do it.
N1.9_Recognition	Text units in this category refer to the attribution of recognition to craftwork.
N1.10_Experiences of socialization and leisure	Text units in this category refer to the attribution of experiences of socialization and leisure to craftwork.
N1.11_Sense of belonging to the territory	Text units in this category refer to the attribution of a greater sense of belonging to the territory to craftwork.
N2_Negative attributions	Text units in this category refer to negative attributions of health, quality of life, and/or well-being to craftwork.
N2.1_Financial instability and labor precarity	Text units in this category attribute labor precarity to craftwork, including multiple causes: low profitability; high production costs and losses; fluctuation in demand; unfair competition; devaluation of craft labor; national economic context; costs of event participation; and lack of social protection.
N2.2_Work overload	Text units in this category attribute the experience of work overload to craftwork.
N2.3_Occupational health problems	Text units in this category attribute the experience of occupational health problems to craftwork.
N2.4_Feelings of dissatisfaction and limitation of creative potential	Text units in this category attribute feelings of dissatisfaction and limitations on creative potential to craftwork, in contrast with the desire for autonomous artistic exploration.
N2.5_Implications of work for personal and social life	Text units in this category attribute negative implications for personal and social life to craftwork.
N2.6_Unfavorable physical conditions and generational rejection	Text units in this category attribute unfavorable physical conditions to craftwork, such as physical discomfort and strong odors, which are perceived as factors contributing to its rejection by younger generations.

N, Node. The gray shading is applied to “parent nodes” (main categories).

Table 5 presents the coding tree with the absolute frequencies of cases and coded references per category and subcategory. To illustrate the corresponding content, excerpts from participants’ statements are included, translated from European Portuguese into English. While minor variations may exist between language versions, none alter the original meaning of the statements.

**Table 5:** Absolute frequencies of cases and coded references by parent and child nodes/categories, with examples of excerpts coded in each category.

Category	Cases	References (N)	Excerpts (examples)
N1_Positive attributions	55	1,235	(See child nodes)
N1.1_Positive implications for psychological well-being and mental health	42	131	“When we work, our brain is always, it’s always working, I mean, it’s an exercise that’s good for our mind and for our health” (artisan, Imagery, 84 years old, male); “Ever since I started doing this craft, my depression has also improved… This gives me life. This gives me life” (artisan, Contemporary Crafts, 67 years old, female).
N1.2_Occupational benefit	33	81	“It only improves, because we have that thing of getting up and coming, otherwise, everyone is lying in bed… and it does not work, so we need to change, to move, in order to have a livelier, more active life” (artisan, Iron and Derivatives, 79 years old, female); “It always affects for the better, it affects because of the pleasure it gives me to occupy my time, which, if I did not have it, would probably be spent more in front of the TV or doing things that were not as interesting” (artisan, Iron and Derivatives, 67 years old, male).
N1.3_Professional fulfillment	50	119	“I really like what I do. I love it, I would not trade clay for anything… It’s a lot of work, but I like what I do” (artisan, Imagery, 88 years old, female); “The artistic activity I engage in, it’s like this, personal well-being. Because I, and I’ll tell you why, the first one to enjoy it, I defend this, the first one to like what we produce has to be ourselves” (artisan, Imagery, 66 years old, male).
N1.4_Realization of creative potential and creative identity	44	209	“Ah, yes, we created, we created designs, because if it were just like befor… now we make those very detailed handmade works. Oh, very, very much” (artisan, Weaving, 71 years old, female); “I learned to develop, and it also has to do with my characteristics, I’m a creative person. I feel good in this creative world, well, it’s a bit of that” (artisan, Imagery, 57 years old, male); “I really enjoy creating. When I’m really in the mood to create, I feel good, I feel good, and I feel that rush. That rush to see the piece completed. It happens to me, but there’s one thing: I get a good kind of anxiety” (artisan, Imagery, 62 years old, male).
N1.5_Evolution and mastery	43	158	“I’m going to tell you what I feel, I never thought in my life, at the age of 88 that I would get this far. I’m telling you this from the bottom of my heart with great joy, thank God, I never thought!” (artisan, Imagery, 88 years old, female); We are always learning, always learning. And it’s that love for learning that we instill within ourselves, that makes us feel good” (artisan, Pottery, 75 years old, male).
N1.6_Sense of generativity and pleasure in teaching	50	211	“we think about it, and I enjoy teaching. When they ask me, I say, you can come to my house and work, and that’s how we teach. My daughter also teaches everything at school, I actually enjoy passing it on” (artisan, Crivo Embroidery of São Miguel da Carreira, 76 years old, female); “I feel like I’m doing something useful for myself and for Barcelos” (artisan, Imagery, 59 years old, female).
N1.7_Economic benefit	35	75	“Look, if I stop, there’s no income because the pension is minimal. And if I stop, when I stop, there’s no income” (artisan, Basketry and Wicker, 75 years old, male); “It’s for us to subsist, with the help of the pension, although the pension is small, but it’s always something” (artisan, Imagery, 76 years old, male).
N1.8_Autonomy and work flexibility	21	50	“Because it’s not done like that, looking at the clock. You understand, it’s when I feel like it and when I’m inspired. And that’s it, now it’s not like a job where I have to show the boss that it’s done, whether it was fast or took little time. It’s the time it takes, it does not matter!” (artisan, Imagery, 71 years old, female); “I’m here working, if I need to go out during the day, I go (…) (artisan, Imagery, 71 years old, female).
N1.9_Recognition	42	155	“I’ve won some awards as well, and those are important for self-esteem and for our activity” (artisan, Imagery, 57 years old, male); “It’s like this: when someone comes up to me and says, ‘Oh, what a beautiful work!’ I come away with my heart full… The best thing they can say to me is, ‘You have some magnificent pieces here.’ That really gives me, it gives me energy to do other things, to make more, to make more and always different” (artisan, Contemporary crafts, 67 years old, female); “And they are still amazed today at how I managed to take the wood, choose it, make everything from start to finish (…)” (artisan, Wood, 62 years old, male).
N1.10_Experiences of socialization and leisure	15	22	“And we make connections, we make connections with the vendors, with each other, and so on” (artisan, Pottery, 78 years old, male); “We have many friends, we go to craft fairs and we all gather there, because many are from far away, and as soon as we get there, it’s like a family” (artisan, Weaving, 71 years old, female).
N1.11_Sense of belonging to the territory	13	24	“My work and that of other artisans may represent a certain value of our country and especially the municipality of Barcelos, as Barcelos is a very important city for craftsmanship in Europe. That’s why it has a title, right, of a creative city” (artisan, Imagery, 84 years old, male); “I like, I like Barcelos, it’s a matter of pride, and even though I do not really like going to craft fair [referring to the Barcelos Craftsmanship and Ceramics Exhibition], I do not like going, but just knowing that I’m showing our skill to those who come from outside…” (artisan, Imagery, 71 years old, female).
N2_Negative attributions	46	344	(See child nodes)
N2.1_Financial instability and labor precarity	37	193	N2.1.1 “(…) this does not bring in as much money as people think it does” (artisan, Imagery, 71 years old, female); N2.1.2 “A bad day is when we open the kiln door and find pieces that have exploded or cracked, because that happens a lot in craft” (artisan, Imagery, 57 years old, female); N2.1.3 “And then there’s also the setback, that we can work for a month or two, sometimes without getting paid, and then everything comes in at once” (artisan, Wood, 62 years old, male).
N2.2_Work overload	21	46	“I’ve been struggling for some time now, as I’m not able to manage the demands of what is asked of me. I’m facing some difficulties. I’ve already had to give up a lot of my Saturdays and Sundays to manage” (artisan, Contemporary crafts, 55 years old, male).
N2.3_Occupational health problems	14	24	“It starts to hurt the spine, and this way of life is very damp for our bones” (artisan, Pottery, 78 years old, male); “then I have my joints in this condition. Everything is done manually” (artisan, Contemporary crafts, 67 years old, female).
N2.4_ Feelings of dissatisfaction and limitation of creative potential	20	49	“We do not have much time to make different things because they do not let us- the customers - they want more, they want their orders, and while we are working on the orders, we are not creating” (artisan, Imagery, 66 years old, male).
N2.5_Implications of work for personal and social life	7	16	Because it takes up so much of my time, I do not have time for anything else, not even to go for a walk, here or there. At least until Easter, I cannot, I just cannot leave” (artisan, Crivo Embroidery of São Miguel da Carreira, 66 years old, female).
N2.6_Unfavorable physical conditions and generational rejection	13	16	“We can never wear nice clothes” (artisan, Imagery, 57 years old, female); “Working with clay is not light work - it’s heavy, quite heavy” (artisan, Pottery, 68 years old, male).

Each excerpt includes the artisan’s attributes: craft and folk art sector, age, and gender.

N, Node. The gray tone is applied to the main categories/“parent” nodes.

#### 3.3.1 Positive attributions (qualitative data)

All participants (*n* = 55, 100%) identified benefits of engaging in craft and folk art activities.

Several subcategories stood out by having emerged in the discourses of more than half of participants: “Professional fulfillment” (N1.3: n_cases_ = 50, n_references_ = 119); “Sense of generativity and pleasure in teaching” (N1.6: n_cases_ = 50, n_references_ = 211); “Realization of creative potential and creative identity” (N1.4: n_cases_ = 44, n_references_ = 209); “Evolution and mastery” (N1.5: n_cases_ = 43, n_references_ = 158); “Recognition” (N1.9: n_cases_ = 42, n_references_ = 155); “Positive impact on psychological well-being and mental health” (N1.1: n_cases_ = 42, n_references_ = 131); “Occupational benefit” (N1.2: n_cases_ = 33, n_references_ = 81); “Economic benefit” (N1.7: n_cases_ = 35, n_references_ = 75). Other subcategories also emerged from participants’ narratives, although mentioned by fewer artisans, including: “Autonomy and work flexibility” (N1.8: n_cases_ = 21, n_references_ = 50); “Experiences of socialization and leisure” (N1.10: n_cases_ = 15, n_references_ = 22); and “Sense of belonging to the territory” (N1.11: n_cases_ = 13, n_references_ = 24).

The subcategory “Professional fulfillment” (N1.3), showing an association between a sense of fulfillment and the artisans’ craftwork, emerged in the discourses of almost all artisans (*n* = 50). The pleasure derived from work, the satisfaction of a job well done, and the pride in seeing the pieces completed stood out as the most expressive ideas, reflecting the importance of craftsmanship for the professional fulfillment it provides: e.g., “It means fulfillment. I think it’s a way for the person who works to feel fulfilled in the works they create. That’s how it’s been over time, what has guided all the people in art to do something, it’s the fulfillment of. It’s a matter of embracing a profession that stays in our soul” (artisan, Imagery, 70 years old, male).

Also frequently referred by the artisans (*n* = 50), was a “Sense of generativity and pleasure in teaching” (N1.6), especially regarding the transmission of knowledge and skills associated to their craft to younger generations, but also to the broader community. The coded references reveal their enjoyment in teaching, their efforts to ensure the continuity of the craft, and a sense of relief in contributing to the preservation of the region’s cultural tradition, as illustrated in the following excerpt: “(…) I feel somewhat relieved, because what I’ve learned I’m teaching someone to carry on the same work, that’s part of it, because it’s a beautiful art” (artisan, Wood, 63 years old, male). There are mentions to the pleasure of seeing their descendants pass on their knowledge to others. Artisans highlight the importance of encouraging the younger generations, starting from childhood, particularly their children and grandchildren, including joint experiences in the atelier, which brings them great satisfaction. There is also the desire to leave their pieces for the younger generations, as well as pride in seeing their family members appreciate their creations. Many mention conducting workshops or training sessions for the community as a rewarding experience for passing on the craft.

The “Realization of creative potential and creative identity” (N1.4) emerged as well a prominent theme among the artisans (*n* = 44). The excerpts highlight the enjoyment of the creative activity, the dedication to new creative challenges, and the exploration of different solutions. They highlight the pleasure in designing unique pieces, according to their own creative sense and freedom, as illustrated by participants’ accounts: “I enjoy always producing something different(…)” (artisan, Imagery, 66 years old, male); “this really means something to me because it was created from the ground up, there’s no material like this, I do not know of any fair, anywhere, where it exists, and I feel good this way” (artisan, Iron and Derivatives, 72 years old, male).

Accompanied by the realization of the creative potential and identity, a sense of “Evolution and mastery” (N1.5), was frequently expressed in artisans’ discourses (*n* = 43). They stressed a sense of growth, both in the preparation of raw materials and in the execution of pieces, reflected in greater technical mastery, improved quality of the pieces, and a pursuit of innovation, as illustrated by the following example, which reflects this sense of evolution and mastery: e.g., “I am aware that over time the work becomes more enriched. Some people think that as the years go by, you lose your skills, but I do not think so. I believe I have a great capacity for creation, just as much, if not more, than when I was younger” (artisan, Imagery, 70 years old, male). There are references to the enjoyment in learning, developing new skills, and of experiencing well-being through the discovery of new details in their craftwork and the observation of their own progress.

In addition to self-recognition of their mastery, “Recognition” (N1.9) by others also emerged prominently in the accounts of a significant number of artisans (*n* = 42). Recognition was associated with a sense of satisfaction derived from honors received at events, invitations from public institutions, and participation in fairs and exhibitions, including international ones. Many artisans mentioned appreciation from clients and the community, highlighting the sense of personal value, happiness, and well-being that such recognition brings them, as expressed by one of the artisans: “I think people treat me very well, and I am happy, I am happy because of that” (artisan, Imagery, 71 years old, female). Recognition from family members and friends was also noted.

Among the most recurrent themes in the artisans’ narratives (*n* = 42, *n* = 131) was the idea of their craftwork having “Positive implications for psychological well-being and mental health” (N1.1). Many participants highlighted the role of crafts in maintaining mental health and psychological well-being, emphasizing various benefits, which are presented below along with examples from their statements. Some participants identified the role of crafts to keep their mind active and stimulating concentration and memory: “This really makes us use our minds; for someone who is almost 72 and still doing this, it’s good for the brain” (artisan, Imagery, 71 years old, female). Many artisans referred to the activity as “therapeutic” and a source of emotional balance, distracting them from negative thoughts and occupying their minds: “It’s a top-level therapy for me… as I say, I come here and forget about illness, forget everything. This is what has helped me. That’s why I like this” (artisan, Imagery, 75 years old, male). Some attributed improvements in depressive symptoms to craftwork, while many highlighted the feelings of happiness, pleasure, and uplift the activity provided: “I think with this I manage to keep my mind healthy, I feel good, I feel happy with that” (artisan, Wood, 57 years old, male). Others mentioned health behavior benefits, such as reduced tobacco consumption or a more active lifestyle: “If I’m not here doing this, I smoke more, and I go back to the TV, which is where I fill the rest of my time” (artisan, Imagery, 84 years old, male). They also associated this involvement with enhanced well-being and health: “It gives me a sense of well-being and satisfaction, and I even think it improves my health” (artisan, Basketry and Wicker, 75 years old, male).

In this line, the idea that craftwork results in “Occupational Benefit” (N1.2) for the artisans was identified by more than half of participants (*n* = 33), who described their activity as a means of remaining active and productively occupying their time. For some, engaging in craft during retirement brought life and joy, as illustrated by the statement: “Because I thought that once I retired everything would be over here, that I’d just cook and wash the dishes, but no, now I feel like my joyful life has just begun” (artisan, Imagery, 71 years old, female). Some participants reported that they would never cease working and would only suspend their craft activity in the event of an incapacitating condition.

Among the practical benefits of the craftwork that were identified by participants, the “Economic Benefit” (N1.7) was frequently mentioned (*n* = 35), often framing that work as a supplement to low retirement pensions, helping them to reach better living conditions, as reported by one participant, for example: “What helps is a bit from the pension, a bit from this, and that’s how we make ends meet, because pensions are small” (artisan, Imagery, 84 years old, male).

In addition, “Autonomy and work flexibility” (N1.8) was stressed as another practical advantage of the craftwork (*n* = 21), concerning the ability of managing their work autonomously, with greater freedom in organizing daily routines and balancing work with personal life. Flexibility was especially valued for enabling artisans to work from home, at their own pace, without rigid schedules, adjusting to their inspiration and existing orders.

Although less frequent, other interesting themes emerging from participants’ discourses included the association of their craftwork to increased “Experiences of socialization and leisure” (N1.10) (*n* = 15), including the opportunities to form friendships during events such as craft fairs, where artisans interact with others in a family-like atmosphere, as one participant noted, illustrating this sentiment: “I love it, I really enjoy socializing with people, because that gives me life, and it’s good to share the good and the bad” (artisan, Contemporary crafts, 67 years old, female). Craftwork activity was seen as promoting social interaction and leisure experiences, including the opportunity to visit new places.

Interestingly, the association of the craftwork to a “Sense of belonging to the territory” (N1.11) was also referred by some artisans (*n* = 13). They expressed pride in belonging to Barcelos and in contributing to the recognition of local crafts as an expression of identity (e.g., the iconic Barcelos Rooster) and national value, with international visibility.

#### 3.3.2 Negative attributions (qualitative data)

Negative attributions (N2) of craftwork to health and well-being also emerged from participants’ discourses, although less expressively (n_cases_ = 46).

The link of craftwork to “Financial instability and labor precarity” (N2.1) stood out (*n* = 37) and accounted for more than half of text segments coded under this category (*n* = 193; 56.10%), with participants stressing the most the low profitability of the work (N2.1.1, *n* = 20) – “This is a beautiful way of life, but in the end, sometimes it’s not compensated, it’s not, it’s not compensated, you know. It requires a lot of work” (artisan, Pottery, 78 years old, male). Besides this, other financial aspects highlighted included the high fluctuation in demand and consequently in their earnings (N2.1.3, *n* = 7); high production costs and losses (N2.1.2, *n* = 6), related to time, materials, process risks, and expenses; “unfair competition” (N2.1.4, *n* = 5), due to the influx of imported industrial products sold at much lower prices; “devaluation of craft labor” (N2.1.5, *n* = 5), often linked to the public’s lack of understanding regarding the time, effort, and costs involved; “costs of event participation” (N2.1.7, *n* = 5), referring to travel, lodging, and registration expenses, frequently without sufficient return; “national economic context” (N2.1.6, *n* = 2), marked by consumers’ limited purchasing power; and “lack of social protection” (N2.1.8, *n* = 2), due to the absence of labor guarantees, stable income, and social rights.

Although fewer in number, some participants also referred to negative consequences of craftwork related to “work overload” (N2.2, *n* = 21), “feelings of dissatisfaction and limitation of creative potential” (N2.4, *n* = 20), “occupational health problems” (N2.3, *n* = 14), “unfavorable physical conditions and generational rejection” (N2.6, *n* = 13), “implications of work for personal and social life” (N2.5, *n* = 7).

Participants who associated their craft activity with work overload (N2.2) (*n* = 21) described working overtime and during non-standard hours - including evenings and weekends - to meet demand, often without additional support.

Participants also mentioned a decline in physical capacity with age, which, combined with the pressure to fulfill orders, results in physical and mental fatigue. Highlighted among these accounts are the following descriptions: “Look, it affects me because at night, I feel very tired, very” (artisan, Wood, 63 years old, male); “Mentally and physically” (artisan, Imagery, 59 years old, female).

Occupational health problems (N2.3) were reported by a small group of artisans (*n* = 14), who complained mostly about musculoskeletal conditions such as osteoarthritis, back problems, and tendinitis, generally attributed to repetitive movements and poor posture, as illustrated by the following excerpt: “All of us who work in handicrafts are like this, we reach a point in life where we have back problems… we all complain about tendinitis, because these are very repetitive movements over many years” (artisan, Imagery, 55 years old, male). Other potentially work-related conditions, including vision and respiratory problems, were also mentioned. The advanced age of the artisans was noted as an additional contributing factor.

A small group of participants (*n* = 13) also highlighted unfavorable physical aspects of the work - such as dirt, strong odors, restrictions on preferred clothing, and outdated work environments - which they associated with younger generations’ rejection of the occupation (N2.6).

In connection with previous aspects such as work overload, a small group of artisans (*n* = 7) highlighted the negative impact of their work on personal and social life (N2.5), including strained family relationships due to demanding schedules or physical absence (e.g., fairs), and difficulties in balancing work, family, and social commitments.

Finaly, a group of artisans (*n* = 20) expressed feelings of dissatisfaction and limitation of creative potential (N2.4), mainly due to commissioned work, often resulting in repetitive tasks and the neglect of personal projects and artistic exploration, as illustrated by the artisan’s statement: “And now there are times when we even want to create, but there are made-to-order pieces, and they take away a bit of the time we need to be creative. To create, we need time, time and inspiration” (artisan, Imagery, 61 years old, female).

## 4 Discussion

This study aimed to understand how older professional artisans perceive the impact of craftwork on their health and well-being. To this end, narratives from artisans across different craft sectors, most of whom engage in this activity as their primary occupation, were analyzed in depth to explore how they attribute mental health, quality of life, and well-being to their work. The study offers an original contribution by examining these topics among older artisans practicing their craft in Barcelos, a UNESCO Creative City of Crafts and Folk Art in Portugal, committed to protecting and promoting its rich cultural heritage and artisanal traditions.

The qualitative data analyzed in this study complement findings from a previous research conducted by the authors (48), providing a richer and more contextualized understanding of participants’ perceptions.

The results highlighted the artisans’ strong engagement with their craft and the important role they attribute to it for their well-being. Most artisans associated their craftwork with a sense of professional fulfillment (N1.3), evolution and mastery (N1.5). These feelings strengthen their connection to the activity and motivate its continuation, regardless of age. Some artisans also reported increased ease in completing complex tasks and greater creativity as they age, countering the perception of age as a limiting factor. These findings are consistent with the literature, which indicates that engagement in the arts later in life contributes to sustained well-being and the slowing of cognitive decline (5). Additional reported benefits include personal growth, the development of physical and cognitive skills, emotional regulation, and enhanced cultural and social awareness (27). Activities such as working with clay, for example, involve intense haptic, proprioceptive, and visual sensations, offering cognitive stimulation through rhythmic movements with positive effects on emotional regulation (23). These results align with studies identifying artistic engagement as an effective strategy to mitigate cognitive decline and promote well-being in healthy populations (5, 8, 9).

The creative potential of older artisans emerged as a central aspect of their experience with crafts and folk art. This was especially evident in the positive attributions, but also appeared in negative ones, underscoring the pervasive role of creativity in the participants’ lives. Craft activity is perceived as a means of realizing creative potential and creative identity (N1.4), associated with the pleasure of creating, satisfaction with the final product, and the well-being derived from the creative act. These findings align with studies linking crafts to positive and meaningful experiences, in which both the creative process and the finished object contribute to well-being (27).

References related to creativity also reflect the quality of the craftsmanship developed by participants, which is consistent with the context of the study site, a UNESCO designated Creative City of crafts and folk art, where local government policies support the value of crafts (52, 53). Literature highlights that context influences the meaning attributed to work (28). Creative motivation emerges as a key factor for sustaining craft practice and artistic growth across the lifespan, contributing to a meaningful life (29). Some participants also expressed a strong sense of belonging to their territory (N1.11), reflected in their pride in local heritage and in the contribution of crafts to the city’s international profile, a sentiment reinforced by this UNESCO designation (52, 53).

However, this creative dimension also revealed tensions. Feelings of dissatisfaction and perceived limitations in their creative potential (N2.4) were often tied to market demands, particularly custom orders and client preferences that restrict artistic autonomy. The challenge of balancing creative freedom with commercial pressures was described by some as “making without creating,” highlighting a desire for more space for personal expression. This tension can be better understood in light of the idea that craft goes beyond manual skills, involving the desire to do a job well for its own sake. Artisans take particular pride in developing skills, which is why mere imitation does not provide lasting satisfaction; the skill must mature (80). This dilemma between creative autonomy and market-driven functional pressures reflects a tension also present in craft work, where self-realization and excellence in making, identified as central dimensions of craft (6), can be limited by market demands. This desire for more space for creative expression can be understood in light of literature showing that late-life creativity is a powerful mechanism of self-fulfillment (81).

Such contradictions reveal not only the constraints experienced, but also the intensity of creative engagement and the importance participants place on autonomy in their artistic work. Previous studies have emphasized the role of craft activities in fostering everyday creativity and lifelong artistic engagement, both contributing to well-being (36). In particular, everyday creativity has been shown to promote positive well-being and general good health (36). This framework is consistent with national priorities, such as Portugal’s Action Plan for Active and Healthy Ageing, which integrates autonomy and independent living, health, and well-being among its strategic pillars (82). Initiatives that support artisans in exploring their creative potential while accommodating the professional demands of their work appear to be of high value. As other studies have argued (81), deepening the understanding of creativity in later life can provide valuable contributions to public policies and to the promotion of active and healthy ageing in a rapidly ageing society. This approach aligns with current international debates and recommendations on cultural and social policies, highlighted by organizations such as the World Health Organization, the United Nations, and UNESCO in the context of creative cities (2, 3, 45–47).

Recognition (N1.9) of the artisans’ work was stressed as positively influencing their personal satisfaction, self-esteem, and social status. Appreciation from family members, clients, fellow artisans, the community, and organizations highlights craftwork as a source of both personal and professional fulfillment. Previous studies have likewise found that the practice of traditional arts and crafts reinforces self-continuity and social status (43). This study, based on a larger and more diverse sample, including different sectors, genders, and both retired and non-retired individuals, supports those conclusions.

This study highlighted the relevance of the economic dimension of craft practice, emerging in both positive and negative attributions. The economic benefit derived from the activity (N1.7) was identified as a central element in participants’ lives, often serving as a supplement to low pensions or even as the primary source of household income. These findings are consistent with previous studies on older populations, which suggest that continued engagement in work is beneficial to health, autonomy, and quality of life, with income being a significant contributing factor (13). In a quantitative study conducted with the same sample, higher income levels from craft activities were significantly associated with more positive perceptions of health and quality of life, further highlighting the importance of income for participants’ subjective well-being (48). The present study reinforces this evidence, offering a contextualized understanding of how participants perceive the impact of craft practice on their well-being. However, economic instability was also strongly emphasized, with the subcategory “Financial instability and labor precarity” (N2.1) standing out as the most prominent among negative references. Several participants reported challenges related to low profitability, high production costs and losses, and fluctuating demand. These conditions contribute to a widespread perception of economic insecurity and are identified as factors discouraging younger generations from engaging in craft. The literature highlights income as a critical determinant of quality of life among older adults (13), and in the Portuguese context, it has been shown to influence both life satisfaction and self-assessed health (41). In this sense, the importance of implementing public policies that promote better economic and working conditions for artisans becomes evident. Such measures are crucial not only for enhancing individual well-being but also for increasing the attractiveness and sustainability of craft activity. The literature on local craft practices recognizes artisans’ right to fair compensation and improved living conditions, viewing craft as a valuable cultural heritage that should be protected and promoted as both a cultural asset and an economic resource for the future (52, 83).

The relevance of these policies is further heightened in the context of global population aging, as they contribute to the autonomy, well-being, and income of this population, ensuring conditions that allow them to remain active and promoting the sustainability of this practice.

Increased funding for creative activities has been identified as an effective strategy for promoting well-being on a broader scale (9). This approach is particularly meaningful when considering intergenerational transmission and engagement of younger community members, as reflected in the sense of generativity observed in this study, which may serve as a key driver in ensuring the continuity of craft practice.

Sense of generativity and pleasure in teaching (N1.6) emerged as central dimensions in this study. Generativity extends beyond the mere transmission of techniques to encompass the sharing of identity and the strengthening of affective bonds between artisans and younger generations (children and grandchildren). Workshops and training sessions aimed at youth and the broader community demonstrate a commitment to the transmission of knowledge and traditions, fostering intergenerational connection and community cohesion. These findings align with literature identifying generativity as a core component of adult development, fostering concern for future generations and correlating positively with life satisfaction (30, 31), and with studies that recognize generativity as a determinant of subjective happiness (84). Furthermore, it is noteworthy that the acquisition of new artisanal skills is possible at all stages of life (37), reinforcing the concept of active aging by acknowledging lifelong learning as a pillar of that process (38–40). This framework further highlights the generative potential of artisans in transmitting their craft, both within the family and in the broader community.

Participants value initiatives aimed at preserving crafts and folk art. In the context of public policies, the implementation of structured knowledge transmission initiatives is suggested, such as mentorship programs between experienced artisans and community members, both young and older, who are interested in craft practice. These initiatives can support the preservation of traditional knowledge, enhance feelings of usefulness, recognition, and personal satisfaction, and help mitigate frustrations related to limitations on creative potential. Given that the limited economic attractiveness of craft activities may hinder the engagement of younger generations, as indicated by the results, such targeted programs become even more essential to promote the continuity of these cultural practices. In analyzing these results and recommendations for practice in light of the mission of the UNESCO Creative Cities Network (UCCN), which aims to strengthen cooperation with and among cities that recognize creativity as a strategic factor for sustainable development, encompassing economic, social, cultural, and environmental aspects (45), this study can serve as a contribution to the global agenda. Thus, the results go beyond the local context, offering insights for other populations, such as other UNESCO creative territories, particularly Creative Cities of Crafts and Folk Art, or communities facing similar challenges, especially in regions that value culture and creativity as drivers of sustainable development. Previous studies highlight that urban life is full of relationships that encourage culture, creativity, and local innovation, and that the UNESCO Creative Cities project seeks to promote these relationships through culture and diversity, fostering the development of cultural groups and enabling local economic growth (85). Other studies have concluded that there is still room to grow and explore innovative and active ways to develop experiences based on the creative industries and intangible cultural heritage (86). Accordingly, the implementation of mentorship programs that pair experienced artisans with community members, including both young and older adult participants, is recommended, fostering the transmission of knowledge and strengthening creative networks. Beyond practice, these programs pave the way for future research on their effects and can be integrated into educational strategies to promote crafts and folk art among new generations. The results reinforce the role of older artisans as guardians and transmitters of traditional knowledge, actively contributing to the preservation of this sociocultural heritage and highlighting their generative contribution. These actions, aligned with UNESCO objectives, help preserve intangible heritage, strengthen community bonds, engage younger generations, and stimulate creative economies. Moreover, these programs can foster social cohesion and promote meaningful engagement of free time, aligning with the United Nations’ Sustainable Development Goals (SDGs) and the guidelines of the World Health Organization (2, 47, 87). Actions grounded in craft practices and folk art not only strengthen the region’s naturally favorable context, characterized by a strong craft identity, but also contribute to enhancing social involvement, community participation, and mental health, which are central components of active and healthy aging (10, 88–90). Some participants noted that the activity gives them autonomy and work flexibility (N1.8), valuing the freedom to manage their own commitments despite the intensive nature of craftwork, free them from rigid schedules, which results in enhanced satisfaction. This finding aligns with the emphasis in the literature on autonomy and the engagement of older adults in meaningful occupations, factors considered fundamental for active aging and contrary to inactivity (39).

The idea that being engaged in craftwork provides opportunities for socialization and leisure experiences (N1.10) emerged among some artisans. While these experiences were valued, they did not appear to be central to daily life, given the intensive dedication to atelier work, which often limited the time available for broader social interactions. Nevertheless, participation in events such as craft fairs was associated with meaningful social experiences, strengthening bonds with colleagues, clients, and fellow artisans. These occasions, beyond their commercial aspect, were perceived as moments of sharing and reunion that contributed to well-being. They held particular significance considering that daily work largely occurred in solitary settings. Social engagement among older adults has been identified as beneficial for mental health and is positively associated with well-being (10). Additionally, a smaller number of participants mentioned implications of work for personal and social life (N2.5), highlighting difficulties in balancing the demands of the craft, such as long working hours or frequent participation in fairs, with family and social engagement. Furthermore, the low profitability of the activity (N2.1), highlighted as a concern by several participants, may compromise the balance between professional and personal life by limiting time spent on recreational and social activities, which can lead to exhaustion and negatively affect artisans’ well-being. These challenges may affect not only artisans’ well-being but also the attractiveness of the profession. The literature has also noted the potential negative impacts of prolonged active life on the subjective well-being of older adults, highlighting the importance of work formats suited to this life stage (12). Mental health promotion and prevention strategies aimed at older populations focus precisely on creating physical and social environments that foster well-being and allow the continuation of meaningful activities (1). The literature has further expanded traditional notions of craft and craftsman, opening new avenues for understanding craftsmanship, creation, and engagement. Artisans particularly value skills that evolve over time, as it is in the slow rhythm of craftwork that practice consolidates and encourages reflection and imagination, elements often incompatible with the pressure for immediate results (80).

The results of this study related to occupational benefit (N1.2) emphasize the relevance of craft activity as a factor for psychological well-being, providing a meaningful and productive occupation. It became clear that staying active through craftwork contributes to a positive perception of time and routine, and it was also highly valued by retirees, for whom the activity represents a meaningful way to fill their days with purpose and enjoyment. The data align with studies that have observed that craft practice improves well-being in various ways, highlighting the creation of a richer, more purposeful life through self-actualization and excellence in craftsmanship (6, 36). The artisans’ statements reflect the positive impact of the activity on psychological well-being and mental health.

Indeed, overall positive implications of craftwork for psychological well-being and mental health (N1.1) emerged as one of the most prominent themes in the participants’ narratives. Beyond this subcategory, other dimensions related to craft practice also reflected perceptions of well-being, suggesting a broad impact on the mental health of artisans. The activity was widely described as promoting feelings of happiness, emotional balance, and motivation, and was often considered therapeutic. Several participants reported that crafting helps them to “clear their minds,” serving as a distraction from worries or health problems, potentially functioning as a relevant coping strategy. Improvements in depressive symptoms were also mentioned, as well as behavioral benefits such as reduced tobacco use, and cognitive stimulation effects, with crafting described as “exercise for the brain. These findings align with literature that highlights the benefits of engaging in creative activities for well-being (7–9), and with studies showing that older adulthood can be a meaningful, productive, and pleasurable phase of life (44). They also converge with research identifying traditional arts and crafts as effective strategies for managing mental health conditions and coping with chronic illnesses (7, 43). The results further corroborate data from a previous quantitative study with the same sample, which identified significant associations between involvement in different craft sectors and benefits in health, quality of life, and well-being (48).

However, some artisans also reported experiences of work overload (N2.2), characterized by long and irregular working hours, including weekends, resulting in physical and mental fatigue. These extensive work periods are often associated with occupational health problems (N2.3), such as musculoskeletal complaints (osteoarthritis, back issues, tendinitis), frequently caused by repetitive movements and improper postures. Deadline pressures and the physical demands of manual work exacerbate these issues, aggravated by the perception of declining physical capacity with age. These challenges may affect not only the physical health of artisans but also compromise the long-term sustainability of the practice by limiting their ability to continue working, which highlights the importance of policies that foster a balanced work environment, promoting artisans’ well-being and health.

The demanding nature of craftwork underscores the need for public policies that promote not only economic recognition and improved working conditions but also a balance between job demands, health preservation, and the encouragement of social relationships. Previous studies, both within the national context involving older adults (41) and with the participants in the current study (48), have linked better economic conditions to more positive perceptions of health, quality of life, and overall life satisfaction, highlighting the importance of ensuring adequate income for artisans’ well-being. Such measures are essential for the sector’s sustainability and for enhancing its appeal to both experienced artisans and new audiences, including younger and older individuals interested in the practice, thereby ensuring the preservation of crafts and folk art as cultural and professional activities.

These recommendations may also be relevant for other contexts that value creativity, such as territories within the UNESCO network. However, it is important to encourage research in these territories to deepen the understanding of the sector’s challenges and opportunities. Such insights will help inform more effective and context-specific public policies, applicable at larger scales, benefiting diverse populations and promoting the health, well-being, and sustainability of crafts and folk art.

Finally, despite the challenges identified, the qualitative results predominantly emphasize positive aspects (N1), highlighting the perceived benefits of the craft activity for artisans’ health and well-being. These findings corroborate quantitative results related to happiness (ESAP), mental health (PHQ-8), and quality of life (WHOQOL-BREF), particularly in the psychological domain (Table 2), and align with studies linking subjective well-being to health and longevity in healthy populations (42). Compared to other studies on the well-being of the Portuguese population (91, 92), the data indicate higher levels of mental health among these participants, corroborating previous quantitative results using the PHQ-8 methodology (48, 92). This body of evidence reinforces the relevance of crafts not only as a form of cultural expression and means of livelihood, but also as a resource that promotes health and psychological well-being.

### 4.1 Future research and limitations

This study makes an important contribution to understanding how older professional artisans perceive the impact of craftwork on their health and well-being. It broadens the scope of artistic activities studied and encourages further comprehensive research in this area (8, 25, 26).

This work offers an original perspective by focusing on ongoing artistic practices developed by older community members outside therapeutic contexts or sporadic experiences. The integration of creative activities, such as traditional crafts and folk art, into public policy represents a strategic opportunity to improve well-being, promote active aging, and preserve cultural knowledge. This study presents several strengths that distinguish it from other work in the field. The multi-methods approach, which integrates quantitative data with an in-depth qualitative analysis through go-along walking interviews conducted in the artisans’ ateliers, provides authenticity and rich contextual insight into participants’ narratives. This relevance is further reinforced by its focus on a UNESCO Creative City of crafts and folk art, connecting local realities with global debates on active and healthy aging, culture, and sustainable development.

It should also be noted that the study was guided by an *a priori* hypothesis of a positive relationship between artistic activity and well-being. While this perspective may introduce potential bias, measures were taken to reduce it, including independent coding by a researcher not involved in the fieldwork and maintaining field notes to ensure critical reflexivity throughout the process.

Future studies using these insights could seek a sufficiently diverse sample, including younger professionals, to allow an intergenerational perspective on these research questions, thereby enriching the sample composition. Furthermore, future studies could explore generativity and the legacy of traditional crafts and folk art, examining their impact on family cohesion and well-being, supported by specific family functioning scales.

The combination of qualitative and quantitative data, grounded in participants’ own attributions, corroborates previous evidence highlighting that craft practice contributes to the psychological well-being and mental health of these artisans. Future research may also benefit from employing both qualitative and quantitative approaches to explore these themes more comprehensively.

These strategies align with the enhancement of the cultural and social status of craft and folk art, recognizing them as noble activities of high cultural relevance and dignity.

## 5 Conclusion

The findings of this study, conducted with older artisans working in crafts and folk art in Barcelos, Portugal, a city recognized by UNESCO as a Creative City of Crafts and Folk Art, enrich the literature by offering a multidimensional perspective on the craft experience in this context, highlighting a particularly positive impact on psychological well-being. Following a multi-method approach that combined quantitative data analysis with the qualitative exploration of individual narratives obtained through walking interviews conducted in the artisans’ ateliers, the study allowed for robust methodological triangulation, supported by independent coding by two researchers and the analysis of both positive and negative attributions emerging directly from the narratives. This set of procedures enhances scientific rigor and distinguishes the study from others that have addressed this population using solely quantitative or qualitative methodologies.

This research makes an original contribution to the field of arts and health by examining how engagement in crafts and folk art is perceived by older artisans as being associated with their health, quality of life, and psychological well-being, reinforcing the value of craft practice beyond mere productivity or occupational engagement.

Despite challenges such as financial instability in the sector, all participants reported predominantly positive impacts from engaging in craftwork, with creativity, mastery, fulfillment, and generativity emerging as key resources for emotional and psychological well-being. In the context studied, craftwork represents more than a mere occupation; it serves as an expression of identity and a culturally valued practice with direct implications for psychological well-being.

In addition to addressing gaps in the literature, the study offers practical recommendations for professionals and policymakers, such as mentorship programs that preserve traditional knowledge, promote the transmission of skills, and encourage younger generations to engage in craftwork through intergenerational collaboration. When integrated into public policies, these measures may contribute to active and healthy aging and reinforce the role of craft as a driver of cultural and economic development. Conducted in a UNESCO Creative City, this study represents a relevant contribution to national and international agendas linking culture, health, and sustainable development, providing insights that, although rooted in a specific context, may inspire other creative settings facing similar characteristics and challenges.

The qualitative data align with previous quantitative findings from the same population, which indicate that artisanal practice was associated with positive perceptions of health and quality of life. These conclusions are particularly relevant in a global context where mental health and psychological well-being are growing priorities.

These results emphasize the value of cross-sectoral actions that integrate culture, health, and social inclusion, grounded in sustainable and identity-based practices rooted in local territories.

In the context of global aging, promoting sustainable activities based on scientific evidence may positively influence public health and cultural policies, particularly regarding active and healthy aging.

## Data Availability

The raw data supporting the conclusions of this article will be made available by the authors, without undue reservation.
