# Supplementary material for: “I feel good in this creative world”: a multi-methods study exploring older artisans’ attributions of mental health, quality of life, and well-being to their work in a UNESCO Creative City of crafts and folk art
Source: Front Public Health. 2025 Oct 2;13:1651932. doi: 10.3389/fpubh.2025.1651932 (PMC12528072; doi:10.3389/fpubh.2025.1651932)
Supplement: Supplementary file 1 [file Data_Sheet_1.pdf]

Consolidated criteria for reporting qualitative studies (COREQ): 32-item checklist

Topic and item no.	Guide questions/description	Reported on page no.
**Domain 1: Research team and reflexivity**		
**Personal Characteristics**		
**1. Interviewer/facilitator**	**Which author/s conducted the interview or focus group?**	The interview was conducted by Author 1 (Sandra Igreja).
**2. Credentials**	**What were the researcher's credentials? E.g. PhD, MD3.**	**Sandra Igreja**<br>PhD Candidate in Gerontology and Geriatrics; MEd, Educational Sciences (specialization in Education and Artistic Expression).<br>**Constança Paúl**<br>Aggregation in Psychology; PhD Biomedical Sciences, expertise in Psychology; Master in Biomedical Sciences; Degree in Psychology.<br>**Soraia Teles**<br>PhD Clinical and Health Services Research;<br>MEcon, Social Economics;<br>MSSc, Health and Clinical Psychology.
**3. Occupation**	**What was their occupation at the time of the study?**	PhD Candidate and municipal officer in social and health services (Department of Education, Health, and Social Services) (SI);<br>Researcher and Full Professor (CP);

		Researcher (ST).
**4. Gender**	**Was the researcher male or female?**	Female
**5. Experience and training**	**What experience or training did the researcher have?**	Experience and training in both qualitative and quantitative data collection and analysis.
**Relationship with participants**		
**6. Relationship established**	**Was a relationship established prior to study commencement?**	No prior relationship with participants.
**7. Participant knowledge of the interviewer**	**What did the participants know about the researcher? e.g. personal goals, reasons for doing the research.**	Participants were provided with a comprehensive information sheet that included the study's aim, participation details, the responsible researchers and contact information (2.2 Participants and recruitment).
**8. Interviewer characteristics**	**What characteristics were reported about the interviewer/facilitator? e.g. Bias, assumptions, reasons and interests in the research topic**	Participants were informed about both the interviewer and the research team (via a comprehensive information sheet), including their names and professional affiliations. The interviewer's interest in the topic stemmed from her academic background and professional experience.
**Domain 2: study design**		
**Theoretical framework**		
**9. Methodological orientation and theory**	**What methodological orientation was stated to**	Multi-methods study with methodological triangulation,

	**underpin the study? e.g. grounded theory, discourse analysis, ethnography, phenomenology, content analysis**	combining quantitative analysis with qualitative content analysis of walking interview narratives (2 Material and methods- 2.1 Study design, 2.4 Data analysis, 2.4.2 Content analysis).
**Participant selection**		
**10. Sampling**	**How were participants selected? e.g. purposive, convenience, consecutive, snowball**	Convenience sampling: artisans aged 55 or older, residing in the community (not in institutional care), and actively working in craft and folk art ateliers located in Barcelos (Portugal) (2 Materials and methods- 2.2 Participants and recruitment).
**11. Method of approach**	**How were participants approached? e.g. face-to-face, telephone, mail, email**	Participants were initially approached by telephone to explain the study, assess preliminary interest, and evaluate eligibility. Those interested in participating were then scheduled for face-to-face data collection, conducted in the artisans' ateliers (2 Materials and methods – 2.2 Participants and recruitment).
**12. Sample size**	**How many participants were in the study?**	55 artisans (3 Results- 3.1 Characterization of the study participants (quantitative data), 3.1.1 Artisans'

		sociodemographic and professional characteristics (descriptive statistics)).
**13. Non-participation**	**How many people refused to participate or dropped out? Reasons?**	Of the artisans who responded to the initial contact, 21 did not participate. Reasons for non-participation included no longer being active in their craft, illness, or not meeting eligibility criteria. None of the participants who agreed to take part dropped out during the study (2 Materials and methods- 2.2 Participants and recruitment; 3 Results- 3.1 Characterization of the study participants (quantitative data), 3.1.1 Artisans' sociodemographic and professional characteristics (descriptive statistics)).
**Setting**		
**14. Setting of data collection**	**Where was the data collected? e.g. home, clinic, workplace**	Data collection was conducted in the participants' ateliers (2 Materials and methods, 2.2 Participants and recruitment).
**15. Presence of non-participants**	**Was anyone else present besides the participants and researchers?**	In general, no. However, during the walking interviews, the presence of other individuals was observed with some participants (from the

		Imagery, Iron and derivatives, and Pottery sectors). These individuals were in different areas of the atelier, engaged in painting activities (e.g., Barcelos Rooster, pottery pieces). Most of them were family members involved in the craft process. Their presence was expected, as family involvement in the craft activity was part of the interview focus and emerged in the results<br>2 Materials and methods- 2.3 Instruments; 3 Results- 3.3 Artisans' attributions of health, quality of life, and well-being to craftwork (qualitative data), 3.3.1 Positive attributions (qualitative data), 3.3.2 Negative attributions (qualitative data).
**16. Description of sample**	**What are the important characteristics of the sample? e.g. demographic data, date**	Sociodemographic characteristics include age, gender, marital status, years of education, professional training, retirement status, main source of income, monthly income, and length of residence in the locality. Professional characteristics include craft sector, age of

		entry into the craft sector, weekly hours dedicated to craft activity, and atelier location (Table 2). Health and quality of life outcomes were measured with the WHOQOL-BREF and PHQ-8 scales, and happiness was assessed using a question from the European Survey on Aging Protocol (ESAP) (Table 3).<br>(3. Results- 3.1 Characterization of the study participants (quantitative data), 3.1.1 Artisans' sociodemographic and professional characteristics (descriptive statistics, 3.2 Artisans' health, quality of life and happiness (quantitative data)).<br>Data collection took place in the artisans' ateliers during the first quarter of 2024 (2 Materials and methods-2.2 Participants and recruitment).
**Data collection**		
**17. Interview guide**	**Were questions, prompts, guides provided by the authors? Was it pilot tested?**	Yes, the questions were prepared by the authors; the interview guide was not pilot tested.<br>The study aimed to explore how older professional

		artisans in Barcelos, a UNESCO Creative City of crafts and folk art, perceive the impact of their activity in crafts and folk art on their health, quality of life, and well-being, identifying positive and negative attributions as well as benefits and challenges.<br>The interview guide included main and secondary questions. The data collection used the "Go-along Walking Interview" method to capture contextualized information within the participants' work settings (2 Materials and methods- 2.3 Instruments).
**18. Repeat interviews**	**Were repeat interviews carried out? If yes, how many?**	Yes, two repeat interviews were carried out to clarify information.
**19. Audio/visual recording**	**Did the research use audio or visual recording to collect the data?**	Yes, the interviews were audio-recorded after obtaining participants' consent (2 Materials and methods- 2.2. Participants and recruitment, 2.3 Instruments).
**20. Field notes**	**Were field notes made during and/or after the interview or focus group?**	Yes, field notes were taken during the walking interviews whenever deemed essential to provide better contextualization and understanding of the

		information collected. This approach aligns with the study's focus on capturing contextual and non-verbal cues to enrich the analysis (2 Materials and methods- 2.3 Instruments).
**21. Duration**	**What was the duration of the interviews or focus group?**	The interviews lasted between 1 and 2 hours (2 Materials and methods- 2.3 Instruments).
**22. Data saturation**	**Was data saturation discussed?**	Yes, data saturation was discussed among the researchers. Saturation was considered to have been reached when no new themes emerged, and the information became redundant.
**23. Transcripts returned**	**Were transcripts returned to participants for comment and/or correction?**	No, transcripts were not returned to participants for comment or correction.
**Domain 3: analysis and findings**		
**Data analysis**		
**24. Number of data coders**	**How many data coders coded the data?**	Two researchers (Researcher A and Researcher B) independently coded all textual data sources (2 Materials and methods- 2.4 Data analysis, 2.4.2 Content analysis).
**25. Description of the coding tree**	**Did authors provide a description of the coding tree?**	Yes. Table 4; 2 Materials and methods- 2.4 Data analysis, 2.4.2 Content analysis; 3

		Results- 3.3 Artisans' attributions of health, quality of life, and well-being to craftwork (qualitative data).
**26. Derivation of themes**	**Were themes identified in advance or derived from the data?**	The themes were derived from the data in an inductive manner.<br>(3 Results, - 3.3 Artisans' attributions of health, quality of life, and well-being to craftwork (qualitative data), 3.3.1 Positive attributions (qualitative data), 3.3.2 Negative attributions (qualitative data).
**27. Software**	**What software, if applicable, was used to manage the data?**	NVivo was used to support content analysis, while SPSS was employed to manage quantitative data and generate descriptive statistics.<br>(2 Materials and methods- 2.4 Data analysis, 2.4.1 Statistical analysis, 2.4.2 Content analysis).
**28. Participant checking**	**Did participants provide feedback on the findings?**	No. (2 Materials and methods- 2.2 Participants and recruitment, 2.3 Instruments)
**Reporting**		
**29. Quotations presented**	**Were participant quotations presented to illustrate the themes/findings? Was each quotation identified?**<br>**e.g. participant number30**	Yes, for both questions. Participant quotations were presented to illustrate the themes and findings. Each quotation was identified with relevant information (e.g.,

		artisan, craft sector, age, gender), such as: (artisan, Imagery, 70 years old, male). See Table 5 and the 3 Results section (3.3 Artisans' attributions of health, quality of life, and well-being to craftwork (qualitative data), 3.3.1 Positive attributions (qualitative data), 3.3.2 Negative attributions (qualitative data).
**30. Data and findings consistent**	**Was there consistency between the data presented and the findings?**	Yes. 3 Results section. There was consistency between the data presented and the findings. Quantitative results were clearly reported and interpreted in line with the study's objectives (3 Results, 3.1 Characterization of the study participants (quantitative data), 3.1.1 Artisans' sociodemographic and professional characteristics (descriptive statistics), Table 2; 3.2 Artisans' health, quality of life and happiness (quantitative data), Table 3. Qualitative findings were supported by illustrative quotations that aligned with the thematic categories identified (Table 5); 3

		Results, 3.3 Artisans' attributions of health, quality of life, and well-being to craftwork (qualitative data), 3.3.1 Positive attributions (qualitative data), 3.3.2 Negative attributions (qualitative data).
**31. Clarity of major themes**	**Were major themes clearly presented in the findings?**	Yes, the major themes were clearly presented in the findings, illustrated with participant quotations and supported by narrative explanations. These themes were also interpreted in the discussion section.<br>3 Results, 3.3 Artisans' attributions of health, quality of life, and well-being to craftwork (qualitative data), 3.3.1 Positive attributions (qualitative data), 3.3.2 Negative attributions (qualitative data), Table 5 (participant quotations, identified by artisan, craft sector, age, and gender).<br>4 Discussion, 4.1 Future research and limitations, 5 Conclusion.
**32. Clarity of minor themes**	**Is there a description of diverse cases or discussion of minor themes?**	Yes. Minor themes and less frequent perspectives were presented in the results and discussed in the

		interpretation. Each category and subcategory emerging from the content analysis was discussed regardless of the frequency or predominance of the responses. This approach allowed the inclusion of less expressive.<br>3 Results, 3.3 Artisans' attributions of health, quality of life, and well-being to craftwork (qualitative data), 3.3.1 Positive attributions (qualitative data), 3.3.2 Negative attributions (qualitative data); 4 Discussion.
